# Focusing on Plates: Controlling Guided Waves using Negative Refraction

**DOI:** 10.1038/srep11112

**Published:** 2015-06-08

**Authors:** Franck D. Philippe, Todd W. Murray, Claire Prada

**Affiliations:** 1 Institut Langevin, UMR 7587 CNRS, ESPCI ParisTech, PSL Research University, 1 rue Jussieu, 75005, Paris, France; 2 Department of Mechanical Engineering, University of Colorado at Boulder, Boulder, CO 80309

## Abstract

Elastic waves are guided along finite structures such as cylinders, plates, or rods through reflection, refraction, and mode conversion at the interfaces. Such wave propagation is ubiquitous in the world around us, and studies of elastic waveguides first emerged in the later part of the 19^th^ century. Early work on elastic waveguides revealed the presence of backward propagating waves, in which the phase velocity and group velocity are anti-parallel. While backward wave propagation exists naturally in very simple finite elastic media, there has been remarkably little attention paid to this phenomenon. Here we report the development of a tunable acoustic lens in an isotropic elastic plate showing negative refraction over a finite acoustic frequency bandwidth. As compared to engineered acoustic materials such as phononic crystals and metamaterials, the design of the acoustic lens is very simple, with negative refraction obtained through thickness changes rather than internal periodicity or sub-wavelength resonant structures. A new class of acoustic devices, including resonators, filters, lenses, and cloaks, may be possible through topography optimization of elastic waveguide structures to exploit the unique properties of backward waves.

Elastic waves that are confined to propagate along a free plate of infinite extent are referred to as Lamb waves. Such waves are dispersive, with the relation between the wave number (*k*) and frequency (ω) given by the well-known Rayleigh-Lamb equation which, at a given frequency, gives a finite number of real, propagating modes within a plate, along with exponentially decaying or evanescent modes^1^,^2^. The Rayleigh-Lamb equation is given by:


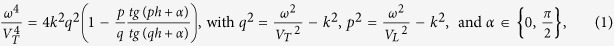


where V_L_ and V_T_ are the longitudinal and shear wave velocities, respectively and *2h* is the plate thickness. The modes are classified as symmetric (α = 0) or anti-symmetric (α = 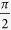) in terms of the plate displacement that is produced with respect to the mid-plane of the plate. The displacement produced by any disturbance to the plate surface can be described by a summation of these modes, with the relative weighting of each mode dictated by the particular forcing function. The form of the dispersion relations depends on both the elastic properties of the plate and the plate thickness. A curious and relatively unexplored feature of Lamb waves is that all propagating modes can exhibit backward wave propagation over some range of Poisson’s ratio, with the exception of the first two anti-symmetric modes A_0_ and A_1_, and the first symmetric mode S_0_. This was proven for isotropic plates by calculating the sign of second order derivative 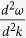 at the origin *k* = 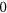 and showing that this curvature is negative for some range of Poisson’s ratio^3^. Note that backward wave propagation is not unique to guided waves in plates, but also exists, for example, for waves guided along rods and cylindrical shells^4^,^5^, and multilayer coatings^6^.

In backward wave propagation, the direction of the energy flux or group velocity is antiparallel to the phase velocity direction, or direction of motion of the phase fronts. Backward wave propagation has seen intense interest in both acoustic and optics of late due to fact that it offers new ways to manipulate acoustic and optical fields and leads to non-intuitive physical effects^7^,^8^,^9^,^10^. It has been demonstrated in engineered acoustic materials through introducing fine scale resonant structures to produce metamaterials^11^,^12^,^13^,^14^,^15^, or by introducing wavelength scale periodicity associated with phononic crystals^16^,^17^,^18^. Backward wave propagation in simple elastic waveguide structures, on the other hand, has seen limited attention. The first experimental demonstration of negative refraction and focusing of Lamb waves, generated through mode conversion between a forward and backward propagating mode, was reported only recently^19^. The result is significant in demonstrating that backward waves in finite structures do, indeed, offer a new means of manipulating elastic waves and can potentially be harnessed to create a new class of acoustic devices. Negative reflection of Lamb waves, associated with mode conversion between forward and backward propagating waves upon reflection, has also been observed^20^.

In this report, we present an efficient and tunable acoustic lens for Lamb waves based on backward wave propagation. The design of the lens is quite simple and consists of a thickness trough introduced into a homogeneous, isotropic duralumin plate as shown in Fig. 1(a). The operation of the lens is as follows: forward propagating waves are generated over the desired frequency range and they mode convert to backward propagating waves at the first thickness step, resulting in negative refraction and focusing within the lens. The backward propagating waves subsequently mode convert back to forward propagating waves at the second thickness step and again undergo negative refraction and focusing at the distal side of the lens. The lens itself is analogous to the flat lens described by Veselago for electromagnetic waves^21^. Here, it is designed to operate on the second symmetric mode (S_2_), with the region outside of the lens supporting forward propagation at the excitation frequency and the lens supporting backward propagation (S_2b_). We note that other modes could be used to create a lens using a similar design, so long as mode conversion between forward and backward propagating waves takes place at the interfaces. The dispersion relation ω(*k*) of the symmetric modes for the plate and lens are shown in Fig. 1(b). The modes are labeled on the plot, with the S_2b_ branch extending from k = 0 to the point at which the slope of the curve goes to zero. The region of interest is shown in the inset where the backward wave in the lens (S_2b_) has the same absolute wave number as the forward wave in the plate (S_2_) at the intersection point of 0.10 mm^−1^.

Using the dispersion relation, the phase velocity in the plate and lens can be found and a simple ray tracing approach, based on Snell’s law, used to predict the focal point within and on the distal side of the lens. Here, we consider rays emanating from the ultrasound source position at the center of the plate edge (Fig. 1(a)) and determine where these rays cross the central plate axis in the lens and plate subsequent to the lens. The results are shown in Fig 1(c), where rays with angles of incidence on the lens of 15, 30, and 45 degrees are considered. Increasing frequency leads to a decrease in the phase velocity in the plate and an increase in the phase velocity in the lens, leading to a shorter focal distance within the lens and a longer focal distance in the plate after the lens. All of the rays converge to the same point within the lens and plate at a coincidence frequency (*f*_*c*_) of 3.32 MHz where the magnitude of the phase velocity in the plate matches that of the lens. Then, geometrically perfect focusing is expected as all rays, regardless of the angle of incidence, converge to the same point. Outside of this frequency, a concentration of acoustic energy is also expected but this will be accompanied by lens aberration; the extent of which increases as one moves further from the intersection point. We also note that the effective aperture of the lens decreases outside of the coincidence frequency as a result of total internal reflection at the lens, further reducing its efficacy.

For efficient operation of the lens, a technique for selective generation of the S_2_ mode in the plate is required. We also desire a point-like excitation source to effectively deduce lens performance. Our approach is to use a 64 element ultrasound array that excites Lamb waves on a strip of material (40 by 10 mm) that has been machined from the plate and is connected to the plate by a sub-wavelength (4 mm) aperture. The array is coupled to the plate through a thin PDMS gel strip. The geometry is illustrated in Fig. 2(a). In order to selectively excite the S_2_ mode, the dispersion relation for the plate is first measured, and the S_2_ mode isolated over the 3.15 to 3.40 MHz range. The dispersion data was then inverse Fourier transformed to yield displacement *u(r, t)* as a function of space *(r)* and time *(t)* for the S_2_ mode. Each transducer element along the excitation strip occupies a different spatial position *(r)* and was driven by a voltage proportional to *u(r, t)* associated with that position (see Supplementary Information S1). The phase delay associated with the dispersive nature of the S_2_ mode is thus captured in the excitation, resulting in preferential excitation and a large enhancement in the strength of this mode with respect to competing modes. The excited pulse propagates through the aperture to the plate, producing a well-defined cylindrical wave pattern.

We now turn to our experimental results demonstrating the operation of the lens. The programmable ultrasound array emits the mode-selected excitation sequence, and the displacement field is measured using a heterodyne interferometer. The interferometer detects the temporal evolution of the normal displacement of the plate surface at each point in the plate and lens. The acquired temporal wave forms are then put through a narrow-band filter to observe the lens behavior within the highly dispersive 3.25–3.5 MHz frequency band. We first demonstrate that the lens does indeed produce negative refraction. The wave forms measured along the centerline (y = 0) from the ultrasound aperture through the lens are filtered at the coincidence frequency 3.32 MHz corresponding to the wavelength at which the phase velocity in the plate is equal in magnitude to the phase velocity in the lens. The displacement amplitude is plotted as a function of the distance x, at regularly spaced instants of time during three periods (Fig. 2(b)). Thus, the waterfall plot shows the displacement amplitudes along the plate through the progression of time. This plot allows one to visually track points of constant phase over time, where a positive slope of such points indicates forward wave propagation and a negative slope indicates backward wave propagation. Forward wave propagation is observed up until the proximal boundary with the lens (x = *18* *mm*) at which time the phase velocity reverses and backward wave propagation is observed. The sign changes again at the distal boundary of the lens (x = *51 mm*) and forward wave propagation is seen on the remainder of the plate.

The ability of the lens to efficiently focus the Lamb wave field is demonstrated by mapping out the displacement fields at several frequencies. Here we present a snapshot of the displacement field measured over the plate at a single instant in time. Figure 3 presents such snapshots over a frequency range of 3.296 to 3.424 MHz. The time domain waveform at a particular frequency is Fourier transformed and the amplitude of the displacement field at a single instant in time determined, yielding an amplitude image over the plate. This image is then spatially low-pass filtered (2^nd^ order Butterworth filter) at a cutoff frequency of 0.18 mm^−1^ to isolate the modes of interest. As expected, the focal point within the lens recedes towards the source with increasing frequency while the focal position on the distal side of the lens increases. The focusing effect of the lens is quite dramatic, particularly within the lens. We have included several movies of wave propagation in the plate in the Supplementary Information (See S2 for movie details). There appear to be higher losses at the interface at the distal side of the lens, which could be attributed to geometrical imperfections. The high degree of dispersion makes the lens very sensitive to frequency, and offers the ability to control and tune the focal point by making small adjustments to the excitation frequency. We also observe the lens serves as an effective collimator of a cylindrical wave field (the focal length goes to infinity) at an excitation frequency of 3.424 MHz.

We next examine the characteristics of the focal region. The intensity of the elastic wave field, given by the square of the displacement amplitude, is plotted in Fig. 4 (a–g) as a function of frequency. The data is processed in a manner analogous to that in Fig. 3, with the amplitude (and intensity) at each frequency determined. The low pass spatial filter frequency was, however, increased to 0.25 mm^−1^ such that it would not influence the characteristics of the focal zone. A pronounced focus is observed within the lens near the coincidence frequency and a weaker, but clearly apparent, focus is seen at the distal side of the lens. The second focus is also offset from the center position of the plate; likely a result of imperfect parallel alignment of the trough edges. While some aberration is apparent, we expect that the lens will follow standard diffraction theory, with the lateral intensity distribution (*I*) of the focal point given by:


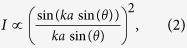


where *k* is the wave number of the S_2_/S_2b_ modes, *a* is the lateral distance and *sin(θ*) is the numerical aperture. Assuming perfect focusing with a numerical aperture of unity and a wavelength of the S_2_/S_2b_ modes of 10.0 mm at the coincidence frequency, this gives a spatial resolution (Rayleigh criteria) of 5.0 mm. The intensity across the focal position inside the lens and on the distal side of the lens are shown in Fig. 4(h, i), respectively, at the coincidence frequency of 3.32 MHz. The solid line in the plots shows the fit to equation (2), with the resolution inside of the lens found to be approximately 5.8 mm and that on the distal side of the lens 6.4 mm. The high numerical apertures achieved (0.85 inside of the lens and 0.78 on the distal side) indicate that effective negative refraction is occurring over a broad range of incident angles. We expect further improvements in lens performance, approaching a numerical aperture of unity, may be possible with a symmetrical thickness change and tighter control of the geometric parameters of the lens.

In conclusion, a simple flat lens using negative refraction has been fabricated: a homogeneous duralumin plate with two parallel thickness changes. The phenomenon occurs within a 7% bandwidth around the coincidence frequency between forward S_2_ and the backward S_2b_ modes. Time domain measurements provide clear evidence of negative phase velocity of the S_2b_ mode within the lens. Focusing close to the Rayleigh diffraction limit was observed within and after the lens. Further studies will investigate the optimization of the plate material and the step height, to maximize the transmission coefficient. New type of acoustic devices, including resonators, filters, lenses, and cloaks, may be possible through topography optimization of elastic waveguide structures to exploit the unique properties of backward waves.

## Additional Information

**How to cite this article**: Philippe, F. D. *et al.* Focusing on Plates: Controlling Guided Waves using Negative Refraction. *Sci. Rep.* **5**, 11112; doi: 10.1038/srep11112 (2015).

## Supplementary Material

Supplementary Video 1

Supplementary Video 2

Supplementary Video 3

Supplementary Information

## Figures and Tables

**Figure 1:**
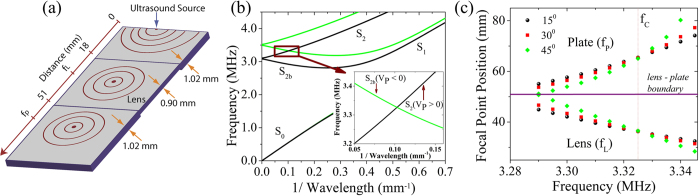
Design of the Lamb wave lens utilizing negative refraction. (**a**) The geometry of the lens. (**b**) Dispersion curves for symmetric modes in the region outside of the lens (thickness = 1.02 mm, black lines) and inside of the lens (thickness = 0.90 mm, light green lines). The inset is a zoom in on the intersection region where the absolute value of the phase velocity (V_p_) is the same in each region of the plate at a spatial frequency of 0.10 mm^−1^. (**c**) The positions at which the rays emanating from the source intersect the central axis of the plate as a function of frequency (color/symbol indicate an incident angle). At the frequency where the phase velocity in the plate is opposite to the phase velocity in the lens (*f*_*c*_) all rays converge to the same point, potentially allowing for geometrically perfect focusing.

**Figure 2:**
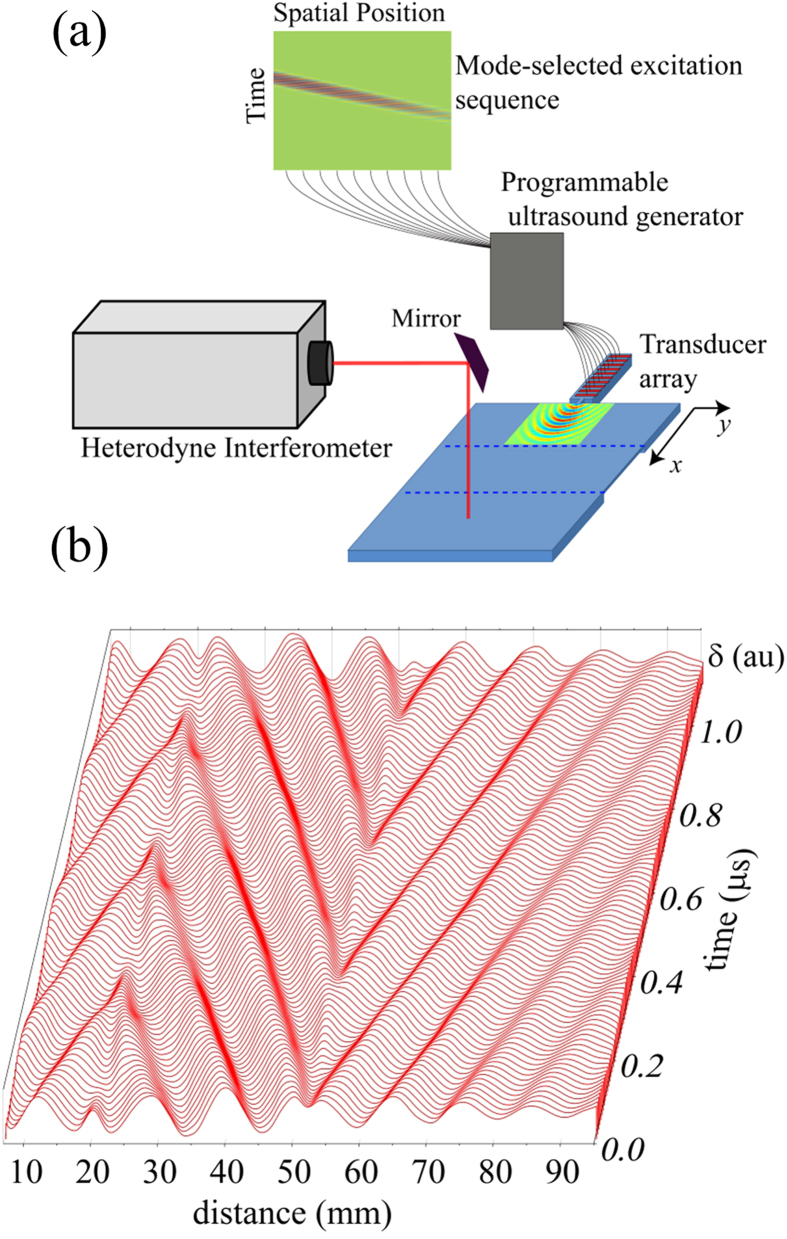
Generation and detection of Lamb waves. (**a**) A transducer array coupled to a programmable ultrasound generator is used to selectively excite the S_2_ mode. The waves are excited on a narrow strip and coupled to the plate through a 4 mm aperture, producing a cylindrical wave pattern. The displacement over the plate is measured using a heterodyne interferometer. (**b**) The temporal evolution of the surface displacement (δ) filtered at 3.320 MHz along the center line in the plate (y = 0). A negative phase velocity is evident within the lens region from 18 to 51 mm.

**Figure 3:**
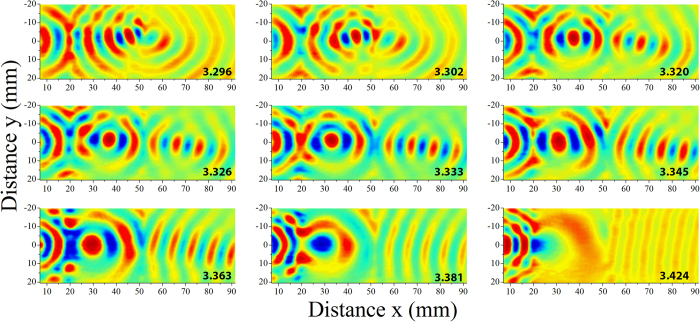
Frequency analysis of the measured field. The temporal displacement is low-pass filtered spatially (k < 0.18 m^−1^) and displayed at a given time for the frequencies indicated on the lower right hand corner of each plot. Within the lens, the focal point moves closer to the proximal interface (at x = 18 mm) as the frequency is increased, while the focal point in the plate recedes and approaches infinity (a collimated beam) at 3.424 MHz.

**Figure 4:**
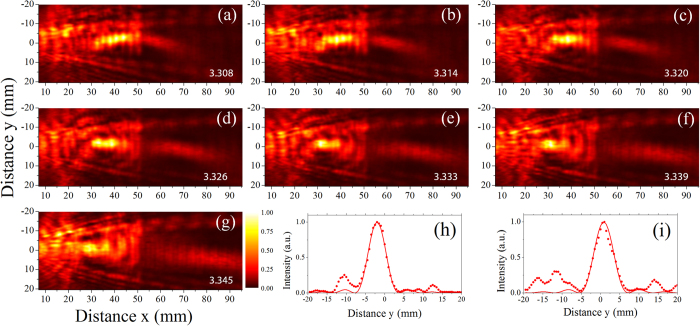
Intensity of the Lamb wave field. (**a** through **g**) The squared amplitude of the displacement field as a function of frequency, where the frequency is indicated on the lower right hand corner of each plot. Focusing of the field is observed in both the lens and plate. The loss of intensity of the focus in the plate may be associated with geometric imperfections at the interface. (**h**) The normalized lateral intensity distribution at a frequency of 3.320 MHz within the lens (at x = 38 mm) and (**i**) the distribution in the plate (at x = 64.5 mm). The dotted lines show the measured data and the solid lines give the best fit to Eqn. 2.
