# Supplementary material for: Focusing on Plates: Controlling Guided Waves using Negative Refraction
Source: Sci Rep. 2015 Jun 8;5:11112. doi: 10.1038/srep11112 (PMC4650665; doi:10.1038/srep11112)
Supplement: Supplementary Information [file srep11112-s4.pdf]

# Supplementary Information

## Focusing on Plates: Controlling Guided Waves using Negative Refraction

Franck D. Philippe[1], Todd W. Murray[2], Claire Prada[1]

*[1] Institut Langevin, UMR 7587 CNRS, ESPCI ParisTech, PSL Research University,*
*1 rue Jussieu, 75005, Paris, France*
*[2] Department of Mechanical Engineering, University of Colorado at Boulder, Boulder, CO 80309*

**S1. Selective generation of the S2 Lamb mode with an array of transducers.**

An array of 64 transducers is used to generate the $S_2$ Lamb mode. The transmitted signals are calculated using a 2D inverse Fourier transform of the selected part of the dispersion curve, *k(f )*. The S2 mode was selected between 3.15 and 3.4 MHz. The transducer array is driven by a Lecoeur Electronics 64-channel programmable device which allows for an 80MHz sampling frequency. The image below shows the amplitude (given by the color scale) of the signal applied to each of the array elements as a function of time. Note that element 0 is the element that is closest to the lens.

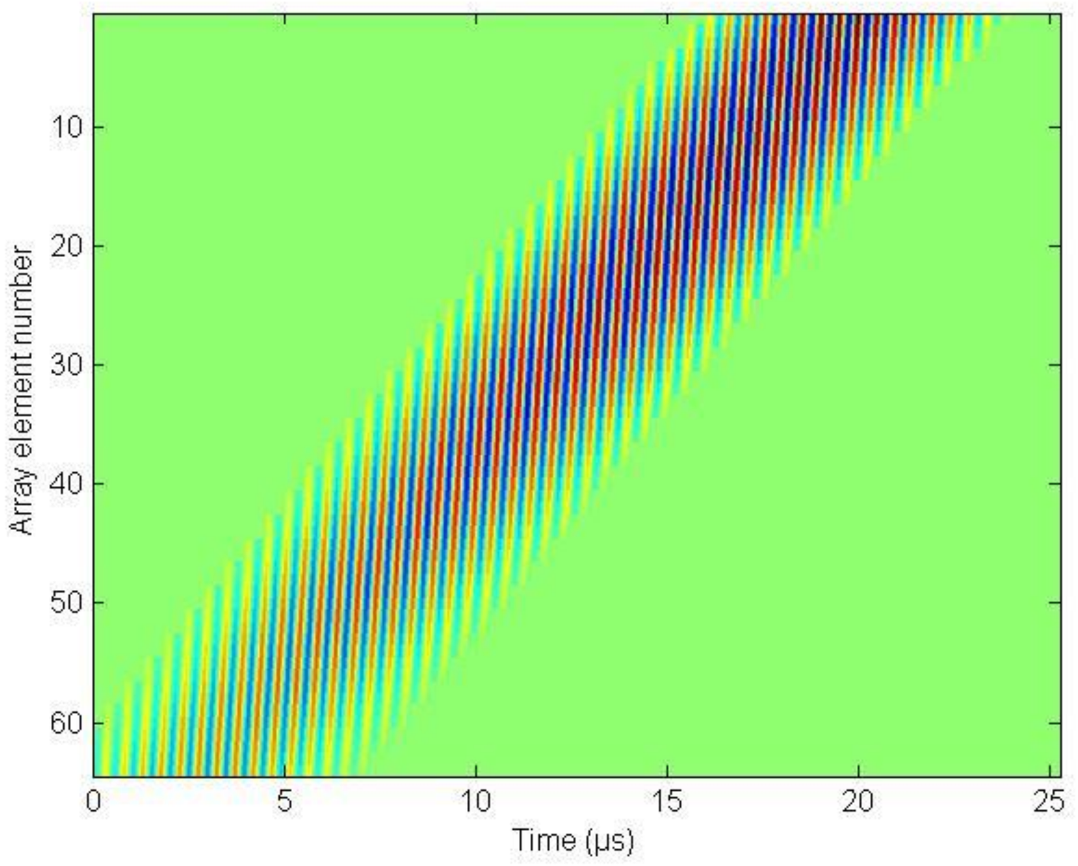

## S2: Movies illustrating the negative refraction and the temporal measurements.

### Supplementary Movie 1

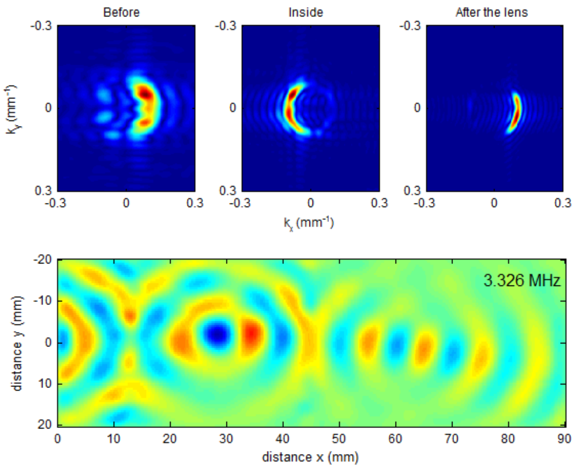


**Spatial frequency analysis**: The 2-dimensional spatial spectrum of the measured displacements is displayed on top and the map of the displacement is displayed below. The spatial spectrum is calculated before the lens (x < 12 mm), within the lens, and after the lens (x > 45 mm). The wave-number domain is limited to $|k_x| < 0.3$ mm$^{-1}$ and $|k_y| < 0.3$ mm$^{-1}$ in order to visualize the conversion of the forward $S_2$ mode ($k_x > 0$) into the backward $S_{2b}$ mode ($k_x < 0$) at the first step and the conversion of $S_{2b}$ mode into $S_2$ mode at the second step. The movie steps through temporal frequencies between 3.20 MHz and 3.45 MHz.

### Supplementary Movie 2

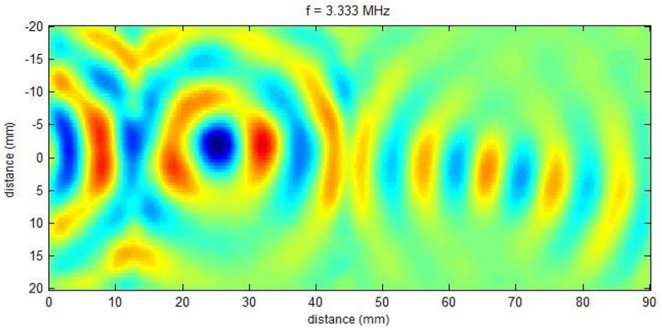


**Single Frequency Surface Displacement:** Movie of the measured surface displacements component at 3.333MHz after spatial low-pass filtering below k = 0.3 mm$^{-1}$ to isolate the $S_2$ and $S_{2b}$ modes. The backward wave and the focusing are clearly observed within the lens, and the field is focused again after the lens.

**Supplementary Movie 3**

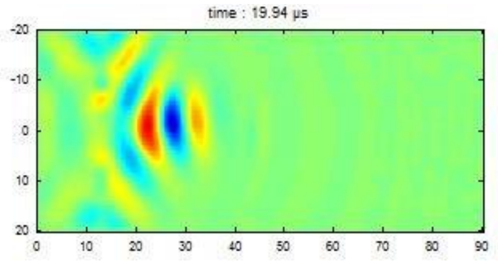


**Broadband Displacement:** Movie of the normal surface displacement measured during 50 μs. The measured signal at each spatial position was band-pass filtered between 3.2 and 3.4 MHz and the images at each time were filtered using a square low-pass filter at a cutoff frequency of $k = 0.3\ mm^{-1}$.
